# Response to Barriers and Facilitators to the International Implementation of Standardized Outcome Measures in Clinical Cleft Practice

**DOI:** 10.1177/10556656211015013

**Published:** 2021-05-11

**Authors:** Conrad J. Harrison, Jeremy N. Rodrigues, Dominic Furniss, Marc C. Swan

**Affiliations:** 1 Nuffield Department of Orthopaedics, Rheumatology and Musculoskeletal Sciences, University of Oxford, UK; 2 Department of Plastic Surgery, Stoke Mandeville Hospital, Buckinghamshire Healthcare NHS Trust, Aylesbury, UK; 3 Spires Cleft Centre, John Radcliffe Hospital, Oxford University Hospitals NHS Foundation Trust, Oxford, UK

**Keywords:** quality of life, psychosocial adjustment, speech disorders

We eagerly read the recent study undertaken by Apon et al. (2021) that describes barriers and facilitators to implementing standardized outcome measurement in cleft care internationally. Specifically, the article considers the implementation of the International Consortium for Health Outcomes Measurement (ICHOM) standard set for cleft lip and/or palate (Allori et al., 2017). The authors surveyed and interviewed cleft care providers from the Netherlands, Sweden, and the United States, before synthesizing common themes within a qualitative research framework. We believe that many themes raised in this paper are generalizable to cleft care in the United Kingdom and Republic of Ireland, based on data gathered as part of the development of the CLEFT-Q computerized adaptive test (CAT).

Cleft care in the United Kingdom and Republic of Ireland is centralized to 12 networked specialist teams located at 18 geographically separate sites. Through this network, we contacted at least 1 clinician from each site to understand departmental barriers and facilitators to implementing the CLEFT-Q scales, which comprise 9 of the 11 patient-reported outcome measures recommended in the ICHOM standard set. Contributions were received from all 18 sites.

Clinicians were asked whether their multidisciplinary team had implemented the CLEFT-Q (and if so which scales) and what their experience of using it was. We enquired whether the questionnaire’s length, the time taken to complete it, and the human resources required to use it were barriers to its implementation. Finally, we asked whether a CAT application that could administer the CLEFT-Q online or via a mobile tablet might facilitate its implementation.

Three of the 12 teams routinely use the CLEFT-Q in clinical practice in 4 of the 18 sites. One team selects scales that are clinically relevant on a per-patient basis, one uses the scales for patients undergoing lip filler injections, and the other uses scales for psychological assessment only.

Our stakeholder engagement exercise corroborates 2 of the major barriers described by Apon et al. (2021): completion time and the “labor-intensive” nature of pen and paper assessment. Burden was considered a barrier to CLEFT-Q implementation in 15 of 18 sites in the United Kingdom and Republic of Ireland. Clinicians at these sites were specifically concerned about the time it takes to complete the questionnaire and how this could impact on clinical workflow. Other concerns related to the human resources required to transcribe results, errors that might occur during the transcription process, and whether results would be available in a timely manner to action in clinic. Clinicians at 16 of the 18 sites felt that the CAT platform would directly facilitate the implementation of the CLEFT-Q at their hospital.

Weidler et al. (2021) explored barriers to standardized outcome measurement in another paper recently published in this journal. In addition to those already described, these authors identify difficulties in getting patients to physically attend follow-up clinics within working hours as a barrier to implementing standardized outcome measurement. Our feedback indicated that the ability to collect CLEFT-Q scores from patients remotely (eg, via a website or smartphone application) would play a major role in its uptake.

An unrelated barrier that emerged from our opinion-seeking exercise that was not described by Apon et al., nor Weidler et al., was the perceived negative wording of items in the speech scales. It is possible that this is a specific barrier to implementing the CLEFT-Q (and therefore the ICHOM standard set) in the United Kingdom and Republic of Ireland. While there is evidence to suggest that patients generally enjoy completing the CLEFT-Q, and specifically its appearance-related items (Klassen et al., 2020), we are unaware of any research that directly addresses the impact of completing the CLEFT-Q speech scales.

We believe that the findings of Apon et al., and Weilder et al., transcend their study samples and are relevant to cleft care in the United Kingdom and Republic of Ireland. The CLEFT-Q CAT (Harrison et al., 2019) will directly address a number of these barriers and facilitate the implementation of the ICHOM standard set, by reducing the time taken to complete CLEFT-Q scales, automating data processing, and accommodating remote data collection as part of the clinical workflow. Further work to address the impact of completing CLEFT-Q speech items would be insightful.
